# Spatiotemporal dynamics of sleep spindles form spiral waves that predict overnight memory consolidation and age-related memory decline

**DOI:** 10.1038/s42003-025-08447-4

**Published:** 2025-07-07

**Authors:** Yiben Xu, Alexander McInnes, Chien-Hui Kao, Angela D’Rozario, Jianfeng Feng, Pulin Gong

**Affiliations:** 1 School of Physics, University of Sydney, Sydney, NSW Australia; 2 CIRUS, Centre for Sleep and Chronobiology, Woolcock Institute of Medical Research, Macquarie University, Sydney, NSW Australia; 3 School of Psychological Sciences, Macquarie University, Sydney, NSW Australia; 4 Institute of Science and Technology for Brain-Inspired Intelligence, Fudan University, Shanghai, China

**Keywords:** Neural circuits, Computational neuroscience

## Abstract

A growing body of evidence has demonstrated that sleep spindles occurring during stage 2 non-rapid-eye-movement (N2) sleep often organize into travelling waves, but the spatiotemporal dynamics of these waves and their functional significance remain unclear. Using high-density electroencephalogram recordings in humans, we demonstrate that N2 sleep spindles frequently form travelling spiral waves, primarily concentrated in the frontoparietal cortices and symmetrically distributed across hemispheres. These spiral waves display rich spatiotemporal dynamics, rotating around phase singularity centers while propagating across the cortex. We find that the propagation trajectories of these spiral waves exhibit two distinct types of behaviour: while some spirals undergo long-range propagation, traversing considerable distances across the cortex, others remain confined to local regions. We illustrate remarkable consistency in the distribution of these trajectories, which repeat across N2 epochs in hours-long recording sessions and remain consistent over a three-month period. Crucially, the consistency of these trajectories can reliably predict subjects’ overnight memory retention performance in a word-pair association task, with greater consistency predicting better performance. Additionally, we find a progressive decrease in trajectory consistency with age, proposing these spiral waves as a potential biomarker for aging. Together, our findings indicate that spiral waves are a defining spatiotemporal feature of N2 sleep and play a crucial role in memory consolidation, offering a promising avenue for further research into sleep-dependent memory processing.

## Abstract (web summary)

Spiral wave patterns of spindle oscillations, a hallmark of large-scale spatiotemporal brain dynamics during sleep, play a functional role in sleep-dependent memory consolidation and show potential as biomarkers of aging.

## Introduction

Sleep spindles, featured by burst-like sequences of 11-15 Hz oscillations, represent a neural hallmark of stage 2 non-rapid-eye-movement (NREM, N2) sleep^1,2^. Over the past decades, extensive research has explored their statistical properties, examining factors such as spindle density, distribution, duration, amplitude and frequency^1,3–9^. While these investigations have yielded valuable insights, linking spindle activities to sleep-dependent memory consolidation^10,11^, intelligence^12,13^, learning abilities^7,14^, brain developments^15,16^ and aging^5,15,17,18^, they often neglect the spatiotemporal organization properties of sleep spindles.

Recent studies have begun to reveal that spindle oscillations, and brain activity in general, are spatiotemporally organized as propagating wave patterns^2,19–22^. Notably, a recent human intracranial electrocorticogram study demonstrated that spindles are often organized into spiral-like rotational wave patterns^2^. Another human study, utilizing intracranial microelectrode recordings, identified wave patterns in spindle oscillations that coordinate phase-locked co-firing sequences of cortical neurons^19^. Despite these discoveries, the organizational principles governing the spatiotemporal dynamics of sleep spindle wave patterns and their functional role remain unclear.

Here, we analyze 256-channel, high-density electroencephalogram (EEG) recordings from patients with obstructive sleep apnea (OSA) during stage 2 NREM (N2) sleep to investigate the spatiotemporal dynamics of sleep spindles. Our analysis reveals that spiral wave patterns, which are organized around phase singularities, are ubiquitous during N2 sleep spindles. These N2 spiral waves, spanning large cortical areas, rotate around phase singularity centers in both clockwise and anti-clockwise directions. Simultaneously, these centers travel across the cortex, organizing spatiotemporal dynamics of sleep spindles. Crucially, we find that the locations and trajectories of these spiral waves remain surprisingly consistent and repetitive throughout the overnight recording session. This consistency persists even across two sessions separated by a three-month interval. Building upon this observation, we introduce a consistency index and demonstrate that a greater index predicts better memory retention performance with high accuracies, providing the first direct evidence of the functional role of these waves in sleep-dependent memory consolidation. In addition, we elucidate that these N2 spirals serve as a potential neural correlate of aging, as evidenced by a significantly reduced consistency index in older subjects. These results thus provide a unique perspective on the neural mechanisms underlying sleep-dependent memory consolidation and the aging-related decline in memory function.

## Results

### Spiral wave patterns underlie spatiotemporal dynamics of sleep spindles

**Fig. 1:**
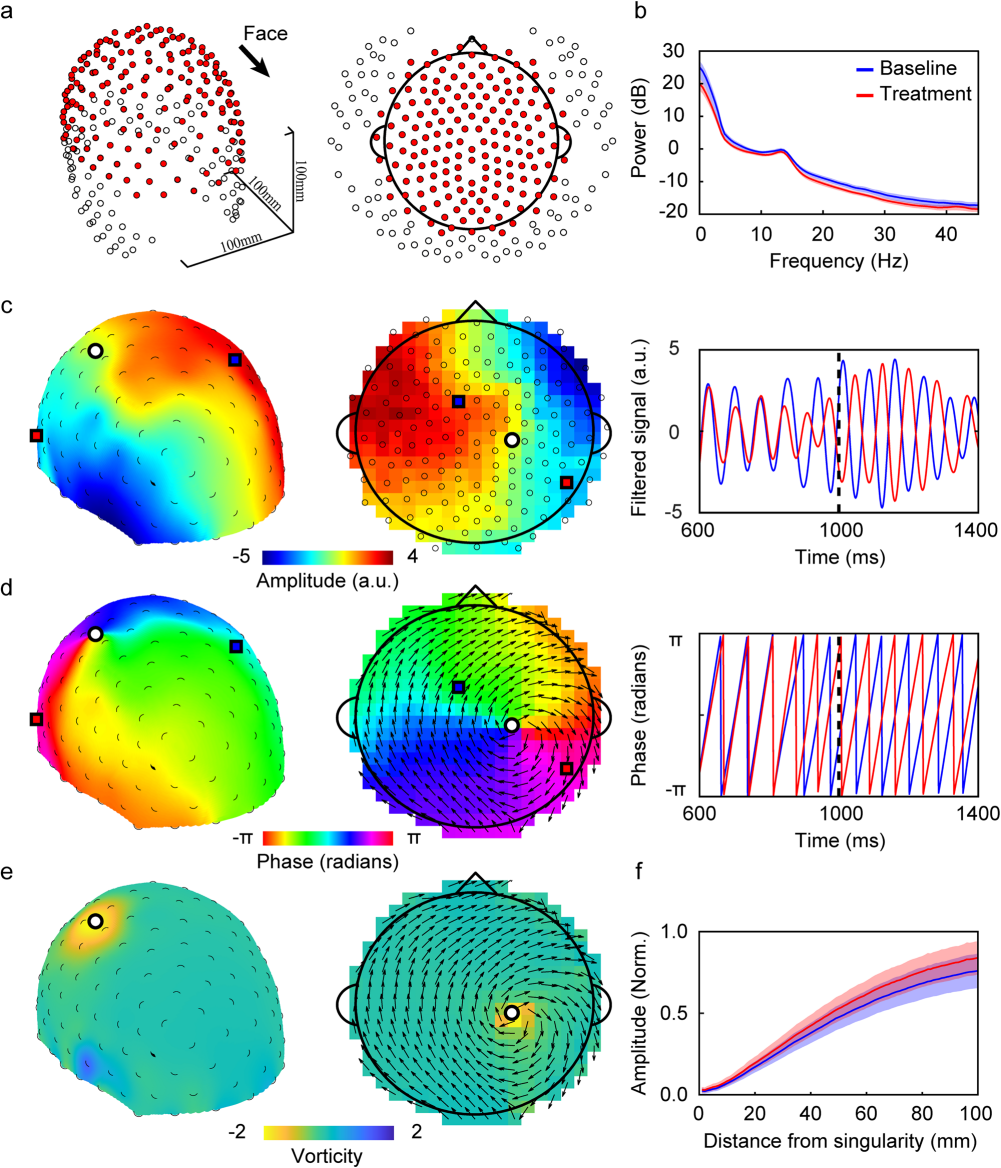
Detection of spiral wave patterns based on moment-by-moment high-density EEG signals. **a** Three-dimensional representation of 256 electrodes (left) and their two-dimensional projections (right). The red dots represent the 166 channels selected for analysis. The black arrow indicates the face direction. **b** Population-averaged power spectrum under the baseline (blue) and treatment (red) conditions. The shaded area denotes the standard error of the mean (s.e.m.), *n* = 9 subjects. **c** 3D spatial distribution of bandpass-filtered EEG signals (11-15 Hz, left) and their 2D projections (middle) at a sample time step marked by the vertical red dashed line (right), along with two exemplar signal time series (red/blue, right) from electrodes marked by squares of corresponding colours. The colour map represents the amplitude of the bandpass-filtered EEG signal. Partially occluded black circles (left) denote the 3D locations of the 166-channel electrode array. Black circles (middle) mark the 2D locations of the 166-channel electrode array. The larger black/white circle denotes the spiral center detected based on the phase velocity field. **d** Similar to **c**, but illustrating the instantaneous phase map (in radians, -π ~ π) at the same time step. Small black arrows depict the phase velocity field. **e** Similar to **c**, but representing the instantaneous vorticity map at the same sample time step. **f** Population-averaged min-max normalized signal amplitude at different distances from detected spiral centers (singularities), under either the baseline (blue) and treatment (red) conditions. The shaded area denotes standard deviation (*n* = 9).

We examine cortex-wide, spatiotemporal dynamics of sleep spindle using 256-channel, high-density EEG recordings from nine patients with obstructive sleep apnea (OSA) going through a continuous positive airway pressure (CPAP) therapy (Methods)^9^. The recordings were conducted across two overnight sessions: one before (‘baseline’) and one after (‘treatment’) the therapy, each lasting approximately 8 hours and separated by around 3 months. To focus our analysis on the cortical regions directly above the brain, we select 166 channels positioned above the ears (Fig. 1a) (Methods), and project the 3D surface formed by these channels onto a 2D topological map (Fig. 1a, Methods). As in existing studies^9,23^, we preprocess the data to identify distinct sleep stages with a focus on stage 2 NREM sleep (N2), which is characterized by prominent spindle oscillations^1^. These oscillations are marked by a distinct peak in the 11-15 Hz frequency range of the power spectrum (Fig. 1b).

To identify spindle epochs, we first apply a second-order Butterworth bandpass filter to extract signal time series within the 11-15 Hz range (Fig. 1c, Methods). We then use an automatic detection algorithm to identify spindle events (Methods)^24^, defining spindle epochs as periods exhibiting such events in one or more channels^25^. Notably, the majority of detected spindle epochs last between 0.5 and 1 second (baseline: 58.53% ± 6.92%; treatment: 58.17% ± 7.28%; mean ± s.d., *n* = 9). The overall mean spindle duration is 1070 ± 74.59 ms (baseline: 1068.00 ± 76.29 ms; treatment: 1072.10 ± 72.88 ms; mean ± s.d., *n* = 9; Fig. S1), which is broadly consistent with previous sleep EEG studies^26^. Within each spindle epoch, we extract the phase time series, φ(x,t), for each channel and produce a 2D phase map at each time step (Fig. 1d, Methods). We calculate the phase velocity field, ${{{{\bf{V}}}}}_{{{{\rm{\varphi }}}}}\left({{{\bf{x}}}},{{{\rm{t}}}}\right)=\nabla {{{\rm{\varphi }}}}\left({{{\bf{x}}}},{{{\rm{t}}}}\right)$^21^ (Fig. 1d, Methods), which allows us to identify different types of wave patterns during spindle epochs^21^. Consistent with a study based on intracranial microelectrode (Utah Array) recordings^19^, we find that around 40% of the phase velocity fields during spindle epochs are organized into spiral wave patterns around phase singularities (Fig. 1d); we refer to these patterns as N2 spirals. As in our recent study^27^, we characterize these spirals by calculating the vorticity values, ${{{{\rm{\omega }}}}\left({{{\bf{x}}}},{{{\rm{t}}}}\right)=\nabla \times {{{\rm{V}}}}}_{{{{\rm{\varphi }}}}}({{{\bf{x}}}},{{{\rm{t}}}})$), as the curl of the phase velocity field surrounding each channel (Fig. 1e). Due to its rotational phase structure, a spiral wave pattern can be characterized by a localized structure of high vorticity values. The sign of the vorticity value indicates its rotational direction, where positive and negative vorticities correspond to anticlockwise and clockwise rotations, respectively. Based on these characteristics, we detect and track N2 spirals (Methods) to study their dynamical and functional properties during spindle epochs.

To statistically examine the significance of spirals detected, we employ a null model with a Fourier-based spatiotemporal phase randomization procedure^27^ (Methods, Supplementary Video 1). This procedure randomizes existing phase structures while preserving the spatial and temporal autocorrelations of the original signals. Spirals identified in the null model exhibit much smaller radii than those found in the original signals; thus, only the spirals from the original signals with a maximum radius equal to or exceeding the 95^th^ percentile of the null distribution are considered significant and retained for further analysis (Methods). In addition, we focus on long-lasting spirals that demonstrate repeated rotations, selecting only those with durations equal to or greater than three rotational cycles (228 ms on average) for further analysis. This is a conservative approach given that the 95^th^ percentile of spiral duration distribution in the null model is 88 ms. We find that N2 spirals (see Supplementary Video 1-4) are consistently present throughout a significant portion of spindle epochs’ duration (baseline: 40.75% ± 1.75%; treatment: 40.82% ± 3.19%, mean ± s.d., *n* = 9), with an average lifespan of 303.23 ± 129.76 ms (mean ± s.d., *n* = 36,212 spiral epochs; baseline: 303.85 ± 129.34 ms, mean ± s.d., *n* = 19256 spiral epochs; treatment: 302.53 ± 130.23 ms, mean ± s.d., *n* = 16,956 spiral epochs). Comparing to spindle epochs, spiral’s lifespan is significantly shorter, such that multiple spirals may be observed consecutively within a single spindle epoch. Although less abundant, we also observe other wave types, such as plane waves (baseline: 11.11% ± 1.80%; treatment: 10.67% ± 2.41%, mean ± s.d., *n* = 9), saddles (baseline: 38.17% ± 4.52%; treatment: 36.93% ± 6.52%, mean ± s.d., *n* = 9), sinks (baseline: 11.47% ± 1.28%; treatment: 11.52% ± 1.18%, mean ± s.d., *n* = 9) and sources (baseline: 13.78% ± 1.40%; treatment: 13.66% ± 1.53%, mean ± s.d., *n* = 9)^21,28^, intertwined with spiral waves (Fig. S2). Notably, regions in proximity to spiral wave centres exhibit a gradual reduction in signal strength, reaching near-zero amplitude at the cores (Fig. 1f), consistent with the hallmarks of phase singularities^27^.

**Fig. 2:**
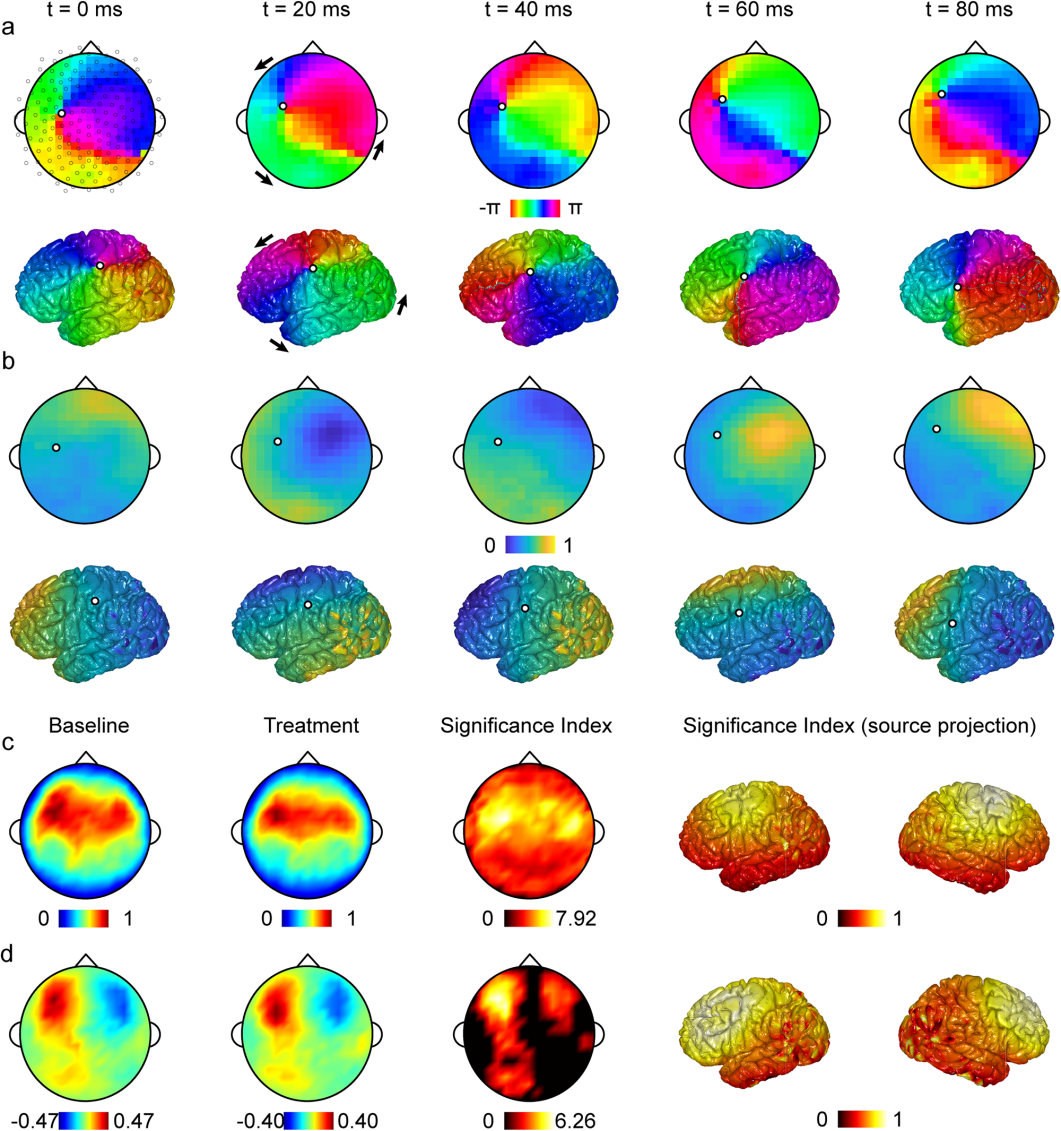
Spatial distribution of spiral waves. **a** Exemplar snap shots of the instantaneous phase field at either the sensor (top) or source (bottom) level. The colour scheme represents the instantaneous phase values (in radians). The black arrows indicate the rotational direction of the spiral wave. The black/white circles denote spiral centre locations. The small grey circles denote the electrode locations projected across the topological map. **b** Same as **a**, but for min-max normalized EEG signal amplitude. Values above and below either top or bottom 1^st^ percentile of the EEG signal are removed as outliers, respectively. **c** Topological map of population-averaged spiral centre density under the baseline (1^st^ column) and treatment (2^nd^ column) conditions, as well as the significance index map at either the sensor (3^rd^ column) or source (4^th^ and 5^th^ columns) level following a significance test against the null model, irrespective of spiral rotational directions. The colormaps in the 1^st^ and 2^nd^ columns represent the min-max normalized spiral centre density, ranging from 0 (minimum) to 1 (maximum). The colormaps in the 3^rd^ column represents the significance index at the sensor level; only regions demonstrating statistically significant indices are shown (significance index > 1.3, equivalent to *P* values < 0.05, *n*  =  9). The colormaps in the 4^th^ and 5^th^ columns represent the min-max normalized sensor-weighted source estimate of the significance index in the 3^rd^ column. **d** Same as **c**, but including spiral rotational directions. The colormaps in the left and middle-left subpanels denote the population-averaged density of extra clockwise (negative, blue) or anticlockwise (positive, red) spirals as a percentage of the maxima in (**c**).

N2 spirals rotate around phase singularity centres that themselves travel across the cortex, as shown in Fig. 2a, b. To characterize the rotational dynamics of N2 spirals, we measure their angular speed, $\varOmega (t)=({\theta }_{t}-{\theta }_{t-\Delta t})/\Delta t$ (Methods). These spirals exhibit consistent angular speeds during rotation, with an average speed of 0.08 ± 0.01 rad/ms (mean ± s.d., *n* = 36,212 spiral epochs; baseline: 0.08 ± 0.01 rad/ms, mean ± s.d., *n* = 19,256 spiral epochs; treatment: 0.08 ± 0.01 rad/ms, mean ± s.d., *n* = 16956 spiral epochs). Due to their large size, these mobile spirals cover a substantial area of the cortex during propagation, potentially playing a crucial role in organizing the large-scale spatiotemporal dynamics of sleep spindles. To illustrate this, we measure the proportion of phase velocity fields across the cortex that are consistent with the rotational dynamics of N2 spirals. On average, 76.06% ± 3.31% (mean ± s.d. *n* = 9; baseline: 75.79% ± 3.05%; treatment: 76.33% ± 3.65%, mean ± s.d. *n* = 9) of phase velocity fields across the entire cortex are consistent with the rotational direction of N2 spirals, supporting their organizational role on a global scale (Methods).

In comparison, we also apply the same procedure to the null model and find only 39.84% ± 0.54% (mean ± s.d. *n* = 9; baseline: 39.80% ± 1.14%, mean ± s.d. *n* = 9; treatment: 39.88% ± 0.74%, mean ± s.d. *n* = 9) of phase velocity fields are consistent with the rotation of N2 spirals, which is significantly lower than in the real signal (*P* < 0.001, *n* = 9). It is worth noting that, while we most often observe single spirals located on one side of the cortex with rotational dynamics extending across both hemispheres, we occasionally observe double spirals coexisting in both hemispheres with opposite rotational directions, albeit less frequently (Fig. S2c).

Although our primary analysis focuses on EEG signal dynamics at the sensor level, it is important to validate these observations at the source level. To this end, we apply a source reconstruction procedure using the Linearly Constrained Minimum Variance (LCMV) beamformer to project the sensor level EEG signal onto the cortical surface (via FieldTrip, Methods). As illustrated in Fig. 2a, b and Supplementary Video 4, the source level projections also exhibit clear spiral waves with spatiotemporal structures that are consistent with those found at the sensor level. These findings thus further validate the presence of N2 spirals and support the robustness of our approach.

### Spatial distribution of N2 spirals

We next investigate the spatial distribution of N2 spirals across the cortex by calculating how many times spiral centers visit individual channel locations during spindle epochs identified throughout N2 sleep (Methods). As shown in Fig. 2c, the population-averaged distributions of N2 spirals, irrespective of rotational direction, are more concentrated in the frontal-parietal cortices under both the baseline and treatment conditions, at both the sensor level and in cortical surface projections. Moreover, a pronounced inter-hemispheric symmetry is evident, with the concentration of spiral centers being well-balanced between two hemispheres (Fig. 2c). To quantify this symmetry, we introduce a similarity index (*r*) representing the spatial correlations between the N2 epoch-averaged spiral distributions of the left and the flipped/mirrored right hemispheres. Consistent with visual inspections, our analysis reveals clear inter-hemispheric symmetry in both the baseline and treatment conditions (baseline: *r* = 0.93 ± 0.03, mean ± s.d.; treatment: *r* = 0.90 $\pm$ 0.03, mean ± s.d., *n* = 9, Fig. 2c). Despite strong inter-hemisphere symmetry in spiral distributions, we also observe clear hemisphericity in terms of local spiral density. To quantify this, we statistically compare the peak local spiral density at individual channels (in significance index, measured by testing the local spiral density at each channel against the null model, Methods) between the left and right hemispheres. Notably, we find significantly higher peak local spiral density in the left hemisphere than the right hemisphere (left: 7.31 ± 3.80, mean ± s.d.; right: 5.04 ± 3.80, mean ± s.d.; *P* < 0.001, *n* = 1000 iterations). However, as this is a local comparison, the observed hemisphericity does not contradict our finding of inter-hemispheric symmetry in spiral distributions, which remains balanced at the global scale. When considering rotational directions, we observe that spiral waves predominantly rotate in temporal-parietal-frontal (TPF) directions (Fig. 2d), which is also reported in a previous human ECoG study^2^. However, this predominance is mostly observed in the bilateral frontal regions, while in the central and parietal regions, spirals are more balanced in both rotational directions. It is worth noting that, this bilateral frontal dominance implies the increased probability of single spirals rotating at TPF directions, rather than the simultaneous presence of two counter-rotating spirals.

### Local and long-range travelling spirals demonstrate distinctive dynamic behaviours

**Fig. 3:**
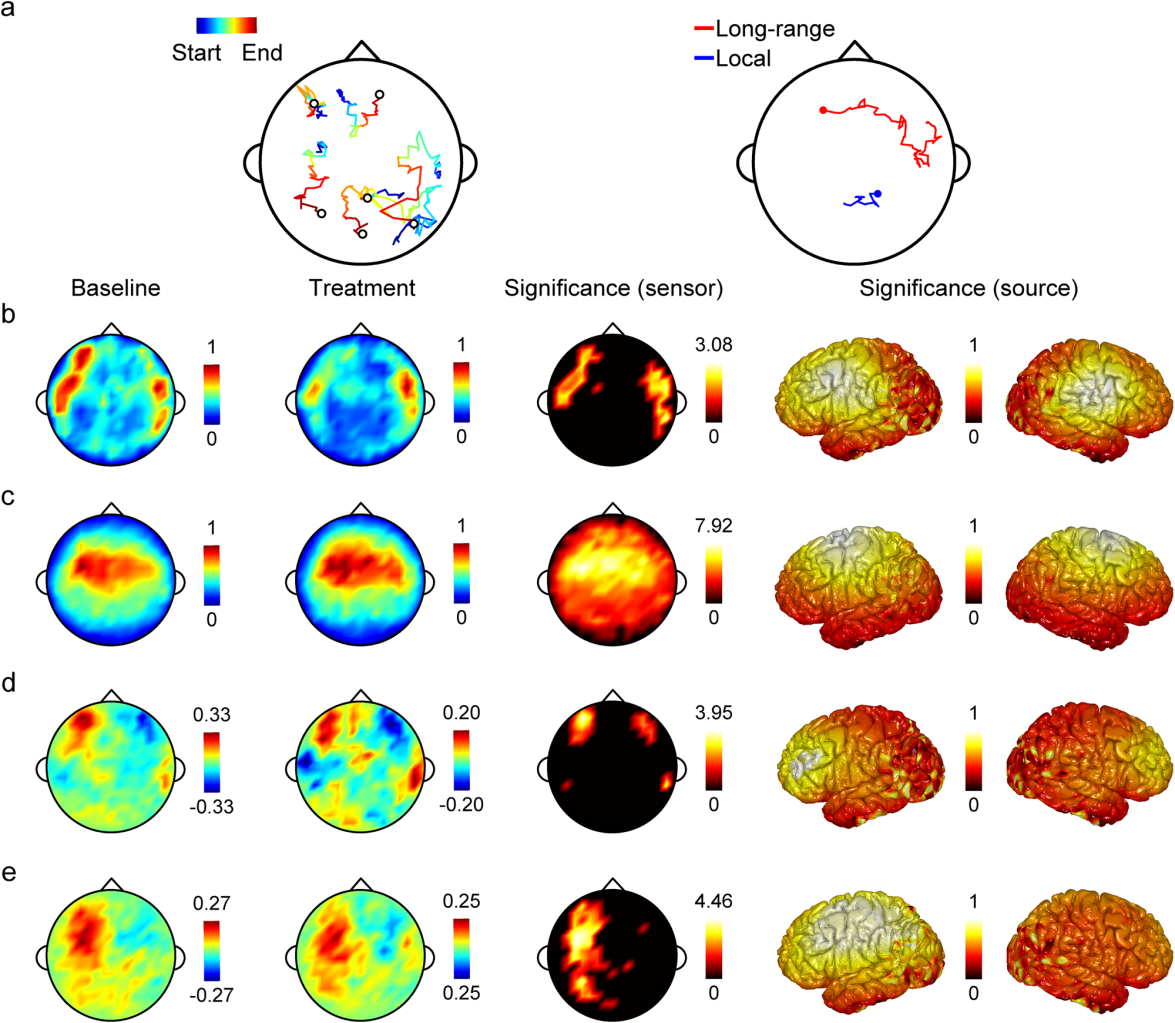
Local and long-range travelling spirals. **a** Left, Exemplar propagation trajectories of spiral centers across the cortex. The colour gradient marks the spirals’ relative positions along their trajectories, from blue (start) to red (end). The black/white circles denote spiral centers at the end of the trajectory. Right, Exemplar propagation trajectories of a local (blue) and long-range (red) spiral center. Blue and red dots denote the corresponding end points of local and long-range spirals, respectively. **b** Topological map of population-averaged spiral centre density of local spirals under the baseline (1^st^ column) and treatment (2^nd^ column) conditions, as well as the significance index map at either the sensor (3^rd^ column) or source level (4^th^ and 5^th^ column) following the comparison between the baseline-treatment-combined spiral centre density map to the null model, irrespective of spiral rotational directions. The colormaps in the 1^st^ and 2^nd^ columns represent the min-max normalized density of spiral centres, ranging from 0 (minima) to 1 (maxima). The colormap in the 3^rd^ column represents the significance index; only regions demonstrating statistically significant indices are shown (significance index > 1.3, equivalent to *P* values < 0.05, *n*  =  *9* subjects). The colormaps in the 4^th^ and 5^th^ columns represent the min-max normalized sensor-weighted source estimate of the significance index in the 3^rd^ column. **c** Same as (**b**), but for long-range travelling spirals. **d** Same as (**b**), but including spiral rational directions. The colour map denotes the population-averaged density of extra clockwise (negative, blue) or anticlockwise (positive, red) spirals as a percentage of the maxima in (**b**). **e** Same as (**d**), but for long-range travelling spirals.

As shown in Fig. 3a, while some spirals are capable of travelling long distances across the entire cortex (Supplementary Video 2), others only jitter around local regions (Supplementary Video 3). Correspondingly, we categorize these spirals into local and long-range travelling spirals according to their maximum travel distances (local spirals: the bottom 10% of spirals by maximum displacement; long-range travelling spirals: the top 10% of spirals by maximum displacement). Compared to local spirals, long-range travelling spirals are featured by significantly longer lifespans (long-range travelling spirals: baseline = 379.08 ± 13.58 ms, mean ± s.d., treatment = 383.02 ± 21.87 ms, mean ± s.d.; local spirals: baseline = 187.76 ± 13.02 ms, mean ± s.d. treatment = 190.57 ± 12.63 ms, mean ± s.d.; false discovery rate/FDR corrected *P* < 0.001 for all pairwise comparisons, *n* = 9 subjects). Although we find no significant difference between the angular speeds of local and long-range travelling spirals (*P* > 0.05 for all pairwise comparisons, *n* = 9 subjects).

More importantly, we consistently observe distinct spatial distributions in local spirals comparing to long-range travelling spirals at both the sensor and source levels, and under both baseline and treatment conditions (Fig. 3b–e). When rotational directions are not considered, long-range travelling spirals are widely dispersed across frontal, central and parietal cortices (Fig. 3c). In contrast, local spirals are predominantly concentrated at the junction of frontal, parietal and temporal lobes in both hemispheres, which aligns well with the location of spiral waves reported in the previous human ECoG study^2^ (Fig. 3b). Similarly, when spiral rotational directions are considered, different distributions of spiral density also emerge between local and long-range travelling spirals (Fig. 3d, e). For local spirals, four distinct clusters with opposite rotational directions can be observed in the bilateral frontal and temporal regions (Fig. 3d). While the frontal clusters predominantly rotate in the temporal-parietal-frontal (TPF) direction, the temporal clusters mostly rotate in the opposite direction (frontal-parietal-temporal, FPT). In contrast, long-range travelling spirals are more widespread and do not exhibit clear bilateral clusters (Fig. 3e).

### Consistency in spiral trajectories predicts memory retention performance

Having illustrated the spatiotemporal dynamics of N2 spirals, we now explore their functional relevance to sleep-dependent memory consolidation, specifically through examining whether the spatiotemporal dynamics of N2 spirals are predictive of the subjects’ overnight memory retention performance in a word pair association task.

As proposed in the two-stage model of memory consolidation^29,30^, the integration and transfer of short-term memory into long-term memory within the neocortex requires the consistent replay of spike sequences related to awake experiences. Aligned with this proposal, recent studies have found that spindle oscillations are organized as precisely repeating spiral waves, with the co-firing of cortical neurons phase locked to their rotational dynamics. These repeating spiral waves thus effectively facilitate the replay of spiking sequences, potentially promoting spike-timing-dependent-plasticity (STDP)^19^. Because the rotational dynamics of these repeating spiral waves are largely determined by the location and polarity of their phase singularity centers, we can effectively quantify the precision of their repetitions by simply measuring the consistency or similarity of their recurring trajectories. In this context, consistent and recurrent spiral trajectories could support more precise replay of neuron co-firing sequences, thus potentially predicting better memory retention.

To validate this hypothesis, we examine whether the consistency of N2 spiral center trajectories can predict subjects’ memory retention performance in a word-pair association task (Methods). First, we categorize the consistency of N2 spiral center trajectories into either short-term or long-term consistency. These are determined by the similarity (via spatial correlations) between spindle-epoch-averaged spiral distributions either from the same overnight recording session (short-term) or from two sessions separated by a three-month CPAP therapy (long-term), respectively (Methods). We then correlate the consistency of spiral center trajectories with the subjects’ memory retention performance of a word-pair association task. In this task, subjects were presented with 32 words (one at a time) and asked to recall their word pairs that were associated previously. This task was conducted pre-/post-sleep and was replicated before (‘baseline’)/after (‘treatment’) a three-month CPAP therapy (see Methods). Note that since long-term spiral consistency effectively measures the ability to maintain a consistent spiral distribution over a three-month period, we specifically investigate its relationship with overall memory performance during this period by averaging memory retention performances across both the baseline and treatment conditions.

**Fig. 4:**
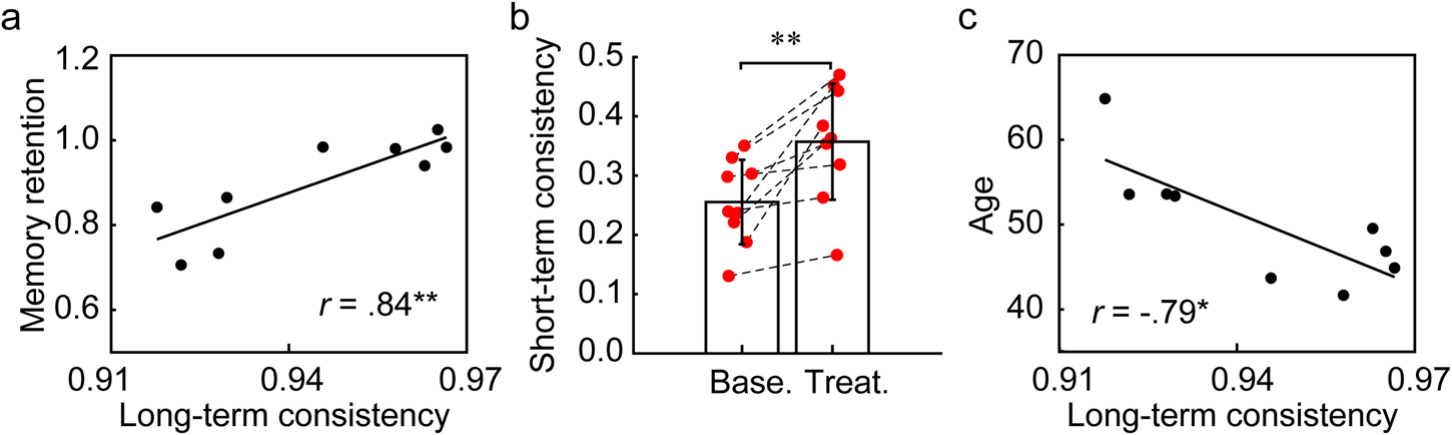
Consistency of N2 spiral centre trajectories predicts memory retention performance and is related to aging. **a** Linear regression showing subject-level spiral long-term consistency against baseline-treatment-averaged memory retention performance (post-/pre-sleep), *n* = 9. Black dots mark the average long-term consistency and memory retention performance (post-sleep memory score divided by pre-sleep memory score) of each subject. * *P* < 0.05, ** *P* < 0.01, FDR corrected for multiple comparisons. **b** Population-averaged spiral short-term consistency under the baseline and treatment conditions. Error bars represent standard deviations (s.d.), *n* = 9. Red dots indicate the mean spiral short-term consistency of each subject. Dashed lines highlight within-subject changes in spiral short-term consistency between baseline and treatment conditions. **c** Linear regression depicting age against subject-level spiral long-term consistency. Black dots represent the mean long-term consistency of each subject at different ages.

As illustrated in Fig. 4a, long-term consistency of spiral distribution is significantly and positively correlated with memory retention performance averaged across baseline and treatment conditions, as measured by the changes in recall performance after sleep (*r* = 0.84, *P* = 0.004, false discovery rate/FDR corrected *P* = 0.008, *n* = 9, Fig. 4a, right), which is considerably higher than correlations found using the conventional methods such as spindle density^23^. Meanwhile, no significant correlation is detected in the null model following the same analysis procedure. Note that, we also find significant correlations between spiral long-term consistency and memory retention performances under the treatment condition (*r* = 0.84, *P* = 0.005, FDR corrected *P* = 0.008, *n* = 9), but not under the baseline condition (*r* = 0.54, *P* = 0.131, FDR corrected *P* = 0.131, *n* = 9). This result is expected, as maintaining a consistent spiral distribution over a three-month period is more likely to impact the memory retention performance at the end of the period (treatment group) than at the beginning (baseline group). In summary, these results support our hypothesis that consistent and recurrent spiral trajectories preserved over a months-long period are critical for sleep-dependent memory consolidation.

We next examine whether the short-term consistency of spiral center trajectories is also related to memory retention performance. Initially, a simple linear regression analysis between spiral short-term consistency and memory retention performance yield no significant correlation. However, by adopting a multiple linear regression approach that considers both short-term and long-term consistencies, we find a notable improvement in our ability to predict the overall recall performances across baseline and treatment conditions. In comparison to the linear regression model based solely on long-term consistency, our multiple regression model shows significantly greater explanatory power (pre-sleep: multiple regression, *r*^*2*^ = 0.91, linear regression, *r*^*2*^ = 0.45, *P* = 0.010, FDR corrected *P* = 0.020, F-test, *n* = 9; post-sleep: multiple regression, *r*^*2*^ = 0.92, linear regression, *r*^*2*^ = 0.57, *P* = 0.010, FDR corrected *P* = 0.020, F-test, *n* = 9). While the multiple regression model shows high explanatory power in predicting memory retention performance (measured as the post-sleep memory score divided by pre-sleep memory score), this effect does not reach statistical significance (multiple regression, *r*^*2*^ = 0.89, linear regression, *r*^*2*^ = 0.71, *P* = 0.082, FDR corrected *P* = 0.082, F-test, *n* = 9), possibly due to the small sample size and the relatively strong performance of the linear regression model. These results thus indicate that while spiral short-term consistency does not significantly predict memory performance on its own, it substantially enhances the prediction of memory performance when combined with long-term consistency. This suggests that spiral short-term consistency is related to memory performance in a conditional manner, likely through interaction or shared variance with spiral long-term consistency.

Taken together, these findings demonstrate that the consistent trajectories of N2 spirals can reliably predict memory retention performance; this underscores the functional significance of N2 spirals in sleep-dependent memory processing.

### Effect of CPAP therapy on spiral consistency and memory retention performance

We next examine the potential impact of the CPAP therapy on both spiral consistency and memory retention performance. As shown in Fig. 4b, we find significant increases in the short-term consistency of spiral distributions following the CPAP therapy (baseline: 0.26 ± 0.07, mean ± s.d.; treatment: 0.36 ± 0.10, mean ± s.d.; *P* = 0.003, paired-samples t-test, *n* = 9). It is worth noting that evaluating the therapy’s effect on the spiral long-term consistency is not feasible, as this process relies on comparing spiral distributions before and after the therapy. On the other hand, we observe no significant change in memory retention following the CPAP treatment (baseline: 0.91 ± 0.15, mean ± s.d.; treatment: 0.89 ± 0.12, mean ± s.d.; *P* = 0.694, paired-samples t-test, *n* = 9), suggesting the impact of CPAP therapy on sleep-dependent memory consolidation remains inconclusive. Considering the non-linear relationship between spiral short-term consistency and memory retention performance observed above, the functional impact of increased spiral short-term consistency following the CPAP therapy also requires further investigations.

### Aging-related decline of spiral long-term consistency

The gradual decline in memory performance is a well-documented aspect of the human aging process^31,32^. Consistently, we find negative correlations between age and declarative memory retention performance across both test conditions, although none reaches statistical significance following FDR correction for multiple comparisons (baseline: *r* = −0.39, *P* = 0.300, FDR corrected *P* = 0.300; treatment: *r* = −0.68, *P* = 0.044, FDR corrected *P* = 0.088; *n* = 9). To examine the relationship between spiral consistency and aging, we correlate the age of each subject with their long-term and short-term spiral consistencies during N2 sleep. As shown in Fig. 4c, we observe a significant negative correlation between age and spiral long-term consistency (*r* = −0.79, *P* = 0.011, FDR corrected *P* = 0.033, *n* = 9), but not between age and spiral short-term consistency (baseline: *r* = −0.10, *P* = 0.792, FDR corrected *P* = 0.834, *n* = 9; treatment: *r* = −0.08, *P* = 0.834, FDR corrected *P* = 0.834, *n* = 9). These results thus suggest that older individuals are more likely to exhibit a decline in spiral long-term consistency, along with reduced memory performance.

One might speculate that the strong correlations between spiral long-term consistency and subjects’ memory retention performance are simply due to their shared associations with age. However, our analysis suggests otherwise. Comparing to age, spiral long-term consistency is a significantly better predictor of memory retention performances averaged across baseline and treatment conditions (long-term consistency Vs. memory retention: *r* = 0.84; age Vs. memory retention: *r* = −0.65; *P* = 0.011, Fisher’s z transformation, *n* = 9), implying a direct relationship that cannot be solely attributed to their shared associations with age. To further test this, we perform multiple linear regression analysis using both the age and spiral long-term consistency as independent variables and memory retention as the dependent variable. We find that including age in the model leads to no increase to the model’s explained variance in memory retention performance (*r*² = 0.71). This suggests that age contributes negligibly on its own, and that its predictive value is largely redundant with spiral long-term consistency (Δ*r*² = 0.05E-2). Conversely, the unique variance explained by spiral long-term consistency is also significantly decreased after adjusting for age (*r*² = 0.28), indicating substantial overlap between the two predictors. Overall, these findings suggest that while age and spiral long-term consistency share some predictive variance, age contributes little independently. Spiral long-term consistency remains the dominant predictor of memory retention performance.

In summary, the gradual decrease in spiral long-term consistency observed in older subjects supports the potential link between N2 spirals, human aging process, and aging-related decline in memory function.

### Spindle consistency enhanced by N2 spirals predicts improved memory retention and declines during aging

As illustrated in the above sections, we find that consistent distributions of N2 spirals preserved over a months-long period reliably predict improved memory performance. It is worth noting that N2 spirals observed in this study primarily represent the phase component of spindle oscillations. In this context, our findings effectively establish a connection between memory consolidation and the spatiotemporal consistency of spindle phase dynamics. While the amplitude component of spindle oscillations (i.e., spindle events and density) has long been associated with sleep-dependent memory processing^10,11^, further investigation is warranted to determine whether the spatiotemporal consistency of spindle amplitude dynamics might also be linked to memory consolidation.

**Fig. 5:**
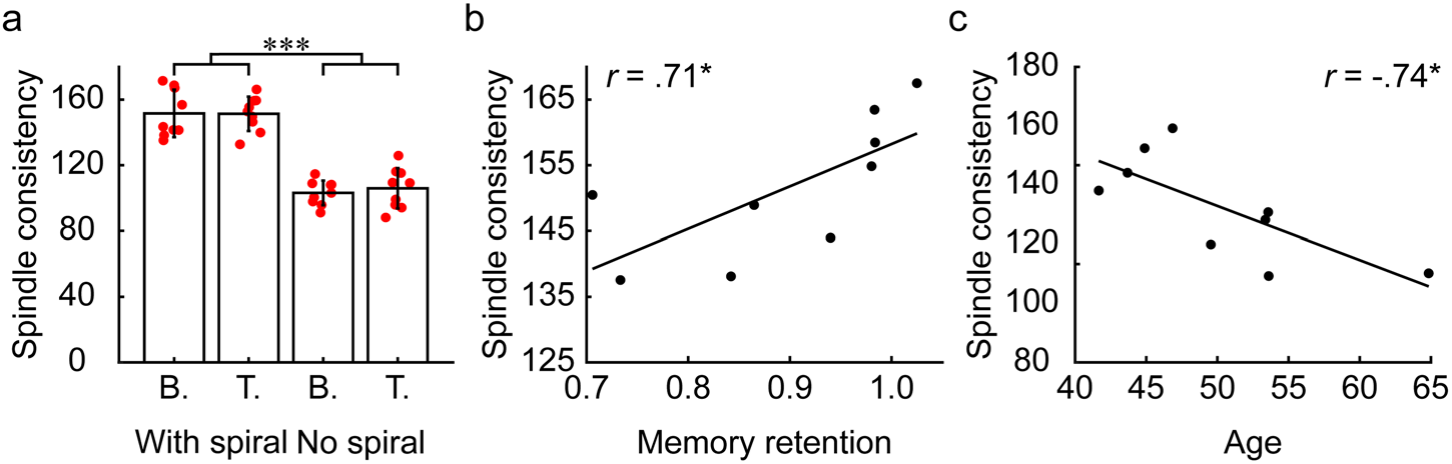
Spindle consistency enhanced by N2 spirals predicts improved memory retention and declines during aging. **a** Spindle consistency with or without the presence of N2 spirals in both baseline (B.) and treatment (T.) conditions, *n* = 9, ***: FDR corrected *P* < 0.001. Red dots represent the mean spindle consistency of each subject. Error bars denote standard deviations (s.d.). **b** Linear regression depicting subject-level spindle consistency against baseline-treatment-averaged memory retention performance in the presence of spirals, *n* = 9, *: FDR corrected *P* < 0.05. **c** Linear regression depicting subject-level spindle consistency against subjects’ age in the presence of N2 spirals, *n* = 9, *: FDR corrected *P* < 0.05.

To test this hypothesis, we adopt a consistency index to measure the spatiotemporal consistency of spindle events across each overnight recording session (Methods) and examine its association with N2 spirals and the subjects’ memory retention performance. As shown in Fig. 5a, we observe a significant increase in spindle event consistency in the presence of N2 spirals compared to their absence (presence of spirals: baseline, 151.60 ± 14.49, mean ± s.d., treatment, 151.37 ± 10.45, mean ± s.d., n = 9; absence of spirals: baseline, 103.11 ± 7.50, mean ± s.d., treatment, 105.90 ± 12.33, mean ± s.d., n = 9, *P* < 0.001, FDR corrected *P* < 0.001 in all pairwise comparisons). Moreover, we observe a significant correlation between the consistency of spindle events and memory retention performance in the presence of N2 spirals (*r* = 0.71, *P* = 0.033, FDR corrected *P* = 0.033, n = 9, Fig. 5b). These findings indicate that spatiotemporally consistent spindle events during overnight sleep are enhanced in the presence of N2 spirals, predicting improved memory retention performance the following morning.

Consistent with the age-related decline of spiral long-term consistency, we observe a gradual reduction in spindle event consistency among older subjects. Similar to their functional relevance, the significant decline of spindle consistency during aging is also observed in the presence of N2 spirals. As illustrated in Fig. 5c, there are significant negative correlations between spindle consistency and subjects’ age in the presence of N2 spirals (*r* = −0.74, *P* = 0.023, FDR corrected *P* = 0.033, *n* = 9), but not when spirals are absent (*P* > 0.05, FDR corrected *P* > 0.05, *n* = 9). This suggests that both the diminished consistency of N2 spirals and spindle events might contribute to age-related declines in memory function, albeit on different time scales. Specifically, while spiral long-term consistency emphasizes the importance of maintaining a stable memory architecture to support the gradual transfer and integration of long-term memories over a three-month period, spindle event consistency underscores the moment-to-moment stability of spindle activity essential for overnight memory consolidation. Given that spindle activity is enhanced in cortical regions involved in encoding^7,33,34^, the spatiotemporal consistency of spindle events likely facilitates the consistent reactivation of neuronal ensembles in regions specific to encoding (via spatial consistency), thereby extending the duration of this reactivation (via temporal consistency).

## Discussion

In this study, we have established that spiral wave patterns of spindle oscillations are a hallmark feature organizing large-scale spatiotemporal brain dynamics during stage 2 NREM sleep. In addition, we have demonstrated direct empirical evidence supporting the functional importance of these spirals in sleep-dependent memory processing and proposed their potential as a biomarker for aging and memory-related disorders.

Previous investigations into sleep spindles have predominantly focused on spindle amplitude-based parameters such as spindle density and distribution^1,3–9^. Our study extends beyond these conventional approaches by systematically characterizing the spatiotemporal dynamics of spindle phase structure. Specifically, we have demonstrated that spiral wave patterns are prevalent during N2 spindle epochs and play a role in organizing the large-scale phase dynamics of sleep spindles. Note that the abundant presence of spiral wave patterns in sleep spindles has been previously documented in human ECoG^2^ and intracranial microelectrode recordings^19^. In line with the ECoG study^2^, we have found large-scale spiral wave patterns spanning multiple cortical areas. This global organization property of spiral waves is consistent with the notion that during sleep, the cortex self-organizes into large-scale coherent states through widespread thalamocortical loops^35^, and that such waves might be particularly relevant for memory consolidation requiring global integration of distributed networks across the cortex. Beyond their global organizational role, we have uncovered a key dynamical property of N2 spirals: they not only rotate around their phase singularities in both clockwise and counterclockwise directions but also propagate across the cortex. These propagations, which can be characterized as local and long-range, exhibit complementary spatial distributions in lateral and central cortical regions, respectively.

Spiral waves have also been identified in our recent study of large-scale brain dynamics in human fMRI recordings^27^. In that study, the abundant presence of spiral-like phase waves was revealed across the cortex of awake human subjects during both resting and task states. Consistent with our observations here, spiral waves found in fMRI recordings also propagate across the cortex with complex trajectories, giving rise to rich spatiotemporal dynamics. Similarly, the spatial distribution of these spirals exhibits clear inter-hemispherical symmetry, as shown in N2 spirals. However, in contrast to N2 spirals, those found in fMRI recordings manifest as more numerous and localized wave patterns. Moreover, they frequently engage in interactions with one another, suggesting distinct mechanisms governing spiral wave dynamics in different brain states (sleep vs. awake). Aside from these large-scale, macroscopic spiral waves, it is worth noting that spirals have been found at the mesoscopic level of neural circuits through various measurement modalities^19,28,36–40^. Taken together, these findings suggest that spiral waves are a general property organizing spatiotemporal brain dynamics across multiple scales. Interestingly, the role of these spirals (also known as vortices) in organizing spatiotemporal dynamics mirrors similar phenomena observed in diverse complex physical and biological systems^41–43^. For example, robust spiral waves have been extensively documented in the heart, where they play a key role in organizing cardiac spatiotemporal activity^43–46^.

As demonstrated in our study, despite exhibiting complex and rich spatiotemporal dynamics, the center distribution of N2 spirals display remarkable consistency, recurring across different NREM epochs even when separated by a three-month interval. Notably, this consistency strongly correlates with memory retention performances, providing direct empirical support for the functional role of N2 spirals in sleep-dependent memory consolidation. Comparing to conventional methods that focus on spindle amplitude-based measures, such as sigma power and spindle density^23^, our spiral-based approach more reliably predicts subjects’ memory retention performance. This underscores the importance of N2 spirals in sleep-dependent memory processing, extending beyond the conventional understanding of sleep spindles. Furthermore, compared to the phase similarity-based method used in a previous study^2^, our spiral-based analysis offers distinct advantages by accounting for both regional and travelling spiral wave patterns. This provides a more comprehensive understanding of the rotational dynamics in spindle oscillations.

As proposed by the two-stage model of memory consolidation^29,30^, short-term memory initially encoded by hippocampus requires repeated sequential reactivations of cortical neurons during sleep to be gradually integrated into the existing long-term memory structure in the neocortex. Importantly, a study based on intracranial microelectrode recordings^19^ has revealed that robust phase-locked sequential co-firing of cortical neurons, with delays less than 25 ms, a time scale conducive to the preconditions for STDP, is coordinated by spindle phase waves. In light of this finding, to facilitate the precise replay of co-firing sequences, it is essential to maintain the consistent repetition of spindle wave patterns. Given that the spatiotemporal dynamics of spindle oscillations are largely organized by the location and rotation direction of N2 spirals on a global scale, the consistent and recurring trajectories of these spirals might thus play an organizational role in the cortical integration (or transfer) of short-term memory into long-term memory across distributed cortical regions.

As demonstrated by the significantly enhanced spatiotemporal consistency of spindle events in the presence of N2 spirals, these robust and self-organized rotational dynamics may extend the duration of spindle episodes in encoding-specific regions^7,33,34^. Although the underlying mechanisms remain unclear, it is possible that consistent and repeated rotations coordinated by N2 spirals may facilitate synchronized firing across the corticothalamic loop, thus supporting maintenance of sleep spindles^47^. Given that enhanced spindle activity, encompassing increased duration, power, and density, is known to improve hippocampus-dependent memory processes^1,3–9^, it is thus plausible that N2 sleep spirals serve as a key moderator in the relationship between spindles and sleep-dependent memory consolidation.

Similar to the observation that spindle density, amplitude, and duration decrease with aging^5^, we also find a gradual reduction in the consistency of spiral center trajectories and spindle events in older subjects. Given the mutually exclusive spatial relationship between spindle amplitude and N2 spiral centers (featured by near-zero amplitude), spiral centers typically avoid regions with high spindle amplitude (i.e., spindle events) and instead propagate through areas with diminished spindle activity. In this context, spatiotemporally less consistent spindle events seen with aging may increase the variability of the landscape that restricts spiral center movement, leading to decreased consistency in spiral center trajectories. Similarly, decreased sigma power, spindle density, amplitude and duration may further lessen spatial constraints, granting spirals greater freedom to move across the cortex. This increased freedom of spiral movement may, in turn, further decrease the consistency of spiral trajectories, which is detrimental to the precise replays of co-firing sequences during memory consolidation, potentially contributing to the observed decline in memory performance among older subjects. However, caution is warranted in interpreting these associations, as they are based on correlational analysis of a relatively small sample. It is likely that reduced consistency in both spindle phase (N2 spirals) and amplitude dynamics (spindle events) contributes to the age-related decline of memory performance, albeit on different time scales (months vs. seconds). Thus, our study of N2 spirals has provided a novel perspective on the neural mechanisms underlying sleep-dependent memory consolidation and aging-related decline of memory function.

Similarly, the decrease in fast spindle number, density, and amplitude in central and posterior cortical areas during N2 sleep has been associated with age-related neurodegenerative disorders such as dementia in Alzheimer’s and Parkinson’s patients^48,49^. Consistent with aging-related memory decline, the disease-induced reduction in spindle activity might also reduce the spatial constraints on N2 spirals and result in lower spiral consistency, which in turn contributes to the decline in memory functions. It is thus plausible to consider establishing spiral consistency as a new biomarker for the diagnosis of age and memory-related disorders associated with abnormal sleep dynamics in future studies. However, it is worth noting that the current study is constrained by its small sample size (n = 9 subjects); thus, future studies with larger populations can further reinforce these findings.

It is also interesting to note that slow wave oscillations ( < 1 Hz) during NREM sleep have been observed to organize as travelling waves across the cortex^50^. Additionally, these oscillations have been shown to couple with sigma power and spindle activity^1^. Given the mutually exclusive spatial relationship between sigma power/spindle activity and N2 spirals, this suggests that the propagation of slow wave patterns might periodically reshape the spatial constraints on N2 spirals through their modulations of sigma power and spindle activity. Thus, it is also worth exploring whether N2 spirals are coupled to the propagating slow waves in coordinating large-scale brain dynamics in future studies.

## Materials and methods

### Ethical approval declarations

The protocol was approved by the University of Sydney Human Research Ethics Committee (Project 2016/712) and the study was prospectively registered on the Australasian and New Zealand Clinical Trials Registry (ANZCTR) - https://www.anzctr.org.au ACTRN12617000336381. All participants provided written, informed consent prior to participation^9^. All ethical regulations relevant to human research participants were followed.

### Experimental design and data pre-processing

We investigate the overnight high-density EEG recordings of nine male participants (age: 50.4 ± 6.5 years, apnea-hypopnea index/AHI: 51.7 ± 23.5/h, mean ± s.d., n = 9) with moderate-severe obstructive sleep apnea (OSA, AHI > 15). Consistent with a recent study^9^, the participants underwent two sessions of overnight polysomnography with 256-channel high-density EEG before and after 3 months of Continuous positive airway pressure (CPAP) therapy. HydroCell Sensor Nets were used to collect high-density EEG data from a whole-head, 256-channel geodesic EEG system (Electrical Geodesics, Eugene, OR). EEG signals were amplified using NetAmps 300 amplifier and NetStation software (PhilipsNeuro EGI, Eugene, Oregon, USA) with an 8-hour time in bed opportunity (10 pm to 6 am). EEG signals were digitized at 500 Hz with a 16-bit analogue-to-digital converter, and impedances were maintained below 10 kΩ. All data were collected in Woolcock Institute of Medical Research, Sydney, Australia. Questionnaires, neurobehavioral battery, and memory tests were collected in the evening prior to an overnight EEG recording, while memory tests were repeated in the next morning.

In the pre-sleep memory tests, the participants were presented with 32-word pairs and immediate recall was tested for each of these 32-word pairs in three consecutive trials. The number of word pairs successfully recalled from the 3^rd^ trial was recorded as the pre-sleep recall score. Similarly, in the next morning (post-sleep), the same word pairs were presented in a randomized order one hour after the lights were turned on and recall was tested. The same procedure was applied in the two sessions of overnight polysomnography, both before (baseline) and after (treatment) the three-month CPAP therapy. Artifacts and arousals in the EEG signals were identified by EEGLAB and visually examined by sleep experts. EEG signals were averaged for 30 s after removing the artifacts using Fast Fourier Transform with a Hanning filter function, 50% overlapping, and with a frequency resolution of 0.25 Hz. Sleep stages (Wake, N1, N2, N3, and REM) were visually scored in 30 s epochs by sleep experts according to standard criteria^51^.

We focus exclusively on the NREM stage 2 (N2) given the prominence of sleep spindles. We temporally filter the recording data using a second-order Butterworth bandpass filter in both forward and backward directions. We set the cutoff frequency of the low-pass filter at 11 Hz and the high-pass filter at 15 Hz to focus on the frequency range of spindle oscillations. We next isolate spindle epochs via an automatic detection algorithm (for details, see later section ‘Spindle event and epoch detection’) and conduct spatiotemporal analysis within those isolated spindle epochs.

### 3D-2D surface projection and linear interpolation

Since the propagation of electrical signals is non-homogenous through the mediums surrounding the brain, the quality of the signal decreases with an increase of the source-sensor distance. To improve signal quality, we remove the channels located below the ear which are not directly above the brain^52^, reducing the number of recorded channels from 256 to 166.

While the original channel locations of the hdEEG recordings are denoted in 3D coordinates, we map these 3D coordinates onto a 2D plane (via EEGLAB 2024.0) to facilitate the analysis of spatiotemporal pattern dynamics. Following the 3D-2D projection, we round these 2D projected channel coordinates and map them onto a uniform *20 × 21* grid. This procedure thus produces a *20 × 21* 2D array at each time step and a 3D matrix (*20 × 21 × t, in x, y, t* coordinates, respectively) for each N2 epoch. Since only 166 channels are mapped onto the *20 × 21* grid (420 grid cells in total), unoccupied grid cells create gaps between channel locations with no data input which makes it difficult to analyze spatiotemporal dynamics. To fill those gaps, we employ a linear interpolation procedure (‘griddata’ function in MATLAB) to fill-in those unoccupied grid cells based-on the signals recorded from nearby channels. Supporting the robustness of the interpolation procedure, we found no significant impact (*P* > 0.05, independent samples t-test between all pairwise comparisons) on our results with different interpolation ratios.

### Spindle event and epoch detection

Consistent with previous studies^9,23,24^, we apply a thresholding algorithm to detect spindle events in bandpass (11-15 Hz) filtered signals, across each of the 166 channels. We first calculate the root mean square (RMS) at each time step within a moving window of 200 ms, followed by a moving average-based smoothing procedure with a window of 200 ms. A spindle event is detected if the smoothed RMS signal remains above 1.5 standard deviations for a duration longer than 0.5 seconds but shorter than 3 seconds. Note that the 0.5-3 second cutoff range is selected according to established pipelines^9,23,24^ that are widely used, based on empirical EEG features and clinical norms. The onset and offset of each spindle event are marked by the crossing points of the 1.5 standard deviations threshold. Finally, we identify spindle epochs when spindle events are detected in one or more channels. The onset and offset of each spindle epoch are determined by the first onset and last offset of the spindle events across all channels, respectively.

### Spatiotemporal consistency of spindle events

We measure the spatiotemporal consistency of spindle events (or spindle event consistency) in each spindle epoch by comparing the spatial distribution of spindle events between all neighbouring time steps within the epoch. This procedure can be described in Eq. (1):

1 $${DS}\left(i\right)={\left(\frac{{\sum }_{t=1}^{{N}_{i}-1}{\sum }_{x=1}^{X}{\sum }_{y=1}^{Y}{{{\rm{abs}}}}(S_{x,y,t+1}-{S}_{x,y,t})/({S}_{x,y,t+1}+{S}_{x,y,t})}{({N}_{i}-1){XY}}\right)}^{-1}$$

Where ${DS}(i)$ denotes the spindle event consistency of the *i*th spindle epoch, ${N}_{i}$ denotes the duration (in time steps) of the *i*th spindle epoch, *X* and *Y* denote the number of columns and rows of the uniform grid produced by 2D projection and linear interpolation (as described in above sections), respectively, ${S}_{x,y,t}$ is the spindle event marker that marks the detected spindle event at time *t*, in a single channel at row *x* and column *y* of the uniform grid, ${S}_{x,y,t}$ is either 1 if spindle events are detected or 0 if no spindle event is detected.

First, we introduce a spindle event marker at every time step throughout an isolated spindle epoch for each channel. The marker is defined as 1 if a spindle event is detected, or 0 if no spindle event is detected. For each channel, we then calculate the absolute differences between the spindle event marker of each time step and the marker of the next time step, which is then divided by the sum of the spindle event markers of both time steps to measure the single-channel change ratio of spindle event markers. This change ratio is 0 if no change occurs in the neighboring time steps (i.e., ${S}_{x,y,t+1}=1$ and ${S}_{x,y,t}=1$), or 1 if there is a change (i.e., ${S}_{x,y,t+1}=1$ and ${S}_{x,y,t}=0$). Note that we ignore the case where no spindle event is detected in both time steps (i.e., ${S}_{x,y,t+1}=0$ & ${S}_{x,y,t}=0$). Finally, we define the spindle event consistency of each spindle epoch as the inversed mean of single-channel change ratios across all channels and time steps throughout the spindle epoch.

To compare spindle event consistency with and without the presence of N2 spirals, we introduce an iterative matching procedure to ensure an equal sample size of channel-averaged change ratios in both conditions. In each of the 100 iterations, we randomly sample time steps from a condition with more time steps within a spindle epoch to match the sample size of the condition with fewer time steps. We then calculate the mean spindle event consistency for each iteration under both conditions before averaging them across iterations to facilitate a comparison between the conditions.

### Phase fields and phase velocity fields

Following the pre-processing steps discussed above, we generate the phase field based on the interpolated grid data. For each channel across the grid, we calculate the instantaneous phases by applying the Hilbert transform. This involves using the ‘Hilbert’ function followed by the ‘angle’ function in MATLAB across the time series of each spindle epoch. The instantaneous phase values of all channels across the 20 × 21 grid thus form a 2D phase field at each time step. Based on these phase fields, we proceed to calculate the phase velocity field using the method developed in our recent study^21^.

### Detection of the core, rotational center and boundary of N2 spirals

Similar to our recent study^27^, based on the phase velocity fields, we proceed to detect spirals by isolating their cores and rotational centers, followed by the defining of their boundary and radius. First, at each time step, we calculate the curl (or vorticity) value of each channel using their neighbouring phase velocity field (using the ‘curl’ function in MATLAB). Next, we apply a filter with a fixed threshold to the curl values (> 1 or < -1) to identify the core of spirals with high vorticity values. This step produces a curl (or vorticity) map with potential locations of spiral cores highlighted by high curl (or vorticity) values. It’s worth noting that we found no significant change in the detection results with moderate variations of the thresholds around -1 and 1.

To detect the core of spirals across space and time, we apply a clustering analysis to the threshold-filtered curl (vorticity) map, which joins and isolates all spatiotemporally connected curl (vorticity) patterns with sizes larger than 3×3 channels. For each spiral with its cores isolated, we then locate their rotational centers by finding the geographic center of each core pattern at each time step.

Once we have located the center of a spiral, we proceed to define its boundary. Here, we adapt the method used in a turbulence study^53^ by modelling the shape of a spiral as circular and fitting a gradually expanding circular surface from the rotational center to its surrounding phase velocity field. Specifically, we measure the angle differences between the phase vectors surrounding the rotational center and the tangent vectors of the expanding circular surface. We then exclude phase vectors with angle differences greater than 45 degrees. The incremental expansion of the circular surface continues until the total number of phase vectors excluded exceeds the radius of the circular surface, which stops the expansion, and the spiral boundary is finalized. To ensure the robustness of the detection algorithm, we also perform manual inspections to verify that the detected spirals are consistent with visual inspection. Note that small variations in either the angle difference or the expansion thresholds have no significant impact on the detection result. Note that, although spiral wave patterns are observed throughout N2 sleep, we only retain N2 spirals found within detected spindle epochs for further analysis.

### Detection of sources and sinks

To detect sources and sinks, we first measure the divergence at each grid location across the phase velocity field at each time step (‘divergence’ function in MATLAB). We then apply a filter with a fixed threshold ( > 1.4 or -1.4) to define the candidate cores of sources (divergence > 1.4) and sinks (divergence < -1.4), followed by a clustering analysis to identify the centers of sources and sinks as the geometric center of these candidate cores. Note that, moderate changes in the divergence threshold do not result in significant alterations in our findings.

Based on the centers detected, we proceed to define the spatial structure of sources and sinks across the grid. To identify the boundary of sink patterns, we first define a starting point at the center of each grid cell, and then iteratively simulate the ‘propagation’ of these starting points across the grid (step size = 0.5 voxels), according to the phase velocity angles linearly interpolated (‘scatteredInterpolant’ function in MATLAB) at their locations at each step of the iteration. The simulated ‘propagation’ stops either when they reach the boarder of the grid or within 1 voxel distance from any detected sink centers, resulting a trajectory starting from each grid locations. Thus, the spatial extent of a full-sized sink pattern can be approximated by the grid cells whose ‘propagation’ trajectories eventually terminate near its center.

To detect source patterns, we simply reverse the propagation directions of the phase velocity field and treat them in the same way as sinks.

Finally, we measure the maximum area (in grid cells) covered by each detected sink and source patterns across their lifespans in comparison to those found in the null model. Only the sinks and sources with maximum areas larger than the 95^th^ percentile of those found in the null model are considered significant and selected for further analysis.

### Detection of saddles

To detect saddle patterns^21^, we first calculate the Jacobian matrix $J\left(x,y\right)$ of the phase vector field $\left({vx},{vy}\right)$ across each voxel of the rectangular grid. This process can be described in Eq. (2):

2 $$J\left(x,y\right)=\left[\begin{array}{cc}\frac{{\partial }{{vx}}}{{\partial }_{x}} & \frac{{\partial }{{vx}}}{{\partial }_{y}}\\ \frac{{\partial }{{vy}}}{{\partial }_{x}} & \frac{{\partial }{{vy}}}{{\partial }_{y}}\end{array}\right]$$

Where $J\left(x,y\right)$ is the Jacobian matrix at the location $(x,y)$, which escribes how the phase vector field (${vx}$, ${vy}$) changes locally at each voxel of the grid.

Next, we calculate the eigenvalues of the Jacobian matrix (‘eig’ in MATLAB). Since saddle patterns are featured by the presence of both positive and negative eigenvalues of their Jacobian matrix, we calculate the product of their eigenvalues and select regions where their products are lower than -0.5 as potential saddle patterns. Finally, we adopt a similar cluster analysis to detect the core of potential saddle patterns, and define their geometric centers as the saddle centers. Note that, moderate changes to this eigenvalue threshold do not have significant impact on the detection output.

Based on the saddle centers detected, we define the spatial structure of saddle patterns. First, we select four starting points along the four principal eigenvector directions, which include the forward and backward directions of the two eigenvectors (two starting points each for the stable and unstable manifolds), located 0.5 voxels away from the detected saddle center. Similar to the spatial structure identification in sink and source patterns, we iteratively simulate the ‘propagation’ of these four starting points (step size = 1 voxel) according to the phase velocity angles linearly interpolated (‘scatteredInterpolant’ function in MATLAB) at their locations at each step of the iteration. The simulated ‘propagation’ stops either when they reach the edge of the grid or fail to reach new grid locations in 2 consecutive iterations. The spatial structure of a detected saddle pattern is thus determined by the final trajectories of these four starting points across the grid.

Similar to sinks and sources, we conduct statistical tests for the saddles detected by comparing their maximum areas (across their lifespan) to those found in the null model. Only saddles whose maximum areas exceed the 95^th^ percentile of those found in the null model are selected for further analysis.

### Detection of plane waves

To detect plane waves, we first measure the global complex polar order parameter $\Psi$ of the phase vector field $\left({v}_{x},{v}_{y}\right)$ at each time step. This procedure can be described in Eq. (3):

3 $$\Psi \left({{{\rm{t}}}}\right)={abs}\left(\frac{1}{N}{\sum }_{j=1}^{N}\exp \left(i* {atan}2\left({v}_{y,j},{v}_{{xj}}\right)\right)\right)$$

Where $\Psi \left({{{\rm{t}}}}\right)$ is the global complex polar order parameter of the vector field $\left({vx},{vy}\right)$ at time t, $N$ is the number of grid cells, ${\theta }_{j}={atan}2({v}_{y,j},{v}_{x,j})$ gives the orientation of each vector $j$, and ${e}^{i{\theta }_{j}}=\exp (i* {atan}2({v}_{y,j},{v}_{x,j}))$ maps the direction of each vector onto the unit circle in the complex plane.

Next, we conduct statistical test for the order parameters measured above at each time step by comparing them to those found in the null model. Only the time steps whose order parameters are larger than the 95^th^ percentile of those found in the null model are considered to contain significant plane waves and retained for further analysis. Note that, consistent with the null model, we compute the order parameters within a maximal sized rectangle over the original grid to remove any NaN values at the edge.

It is also worth noting that, since the detection of sources, sinks, saddles and plane waves are not the focus of this study, to save processing time, we randomly select 10% of spindle epochs to conduct above analysis. However, given the large number of spindle epochs detected throughout the overnight recording, this random sampling procedure still yields thousands of epochs for each subject. Therefore, the detected wave patterns are likely robust and representative of the entire recording period.

### Spiral density significance index

We introduce a significance index to evaluate the statistical significance of population-level spiral density distributions against a null model with an iterative random sampling procedure. First, we compute the N2-epoch-avearged spiral density map for each subject under both the baseline and treatment conditions. Next, using two-tailed paired-sample t-tests, we compare the N2-epoch-avearged spiral density distribution (pooled across all subjects and test conditions) at each channel location against the null model (which underwent the same procedure) at a randomly sampled channel location. This procedure is repeated 1000 times with each iteration sampling a random channel location from the null model, thus generating 1000 *P*-values for each channel location. These *P*-values are subsequently averaged to calculate the spiral density significance index for each channel location. This procedure can be described in Eq. (4):

4 $${{SI}}_{j}={-\log }_{10}^{{P}_{j}}$$

where ${{SI}}_{j}$ is the significance index at channel *j*, and ${P}_{j}$ is the iteration-averaged *P*-value of spiral density tested against the null model at channel *j*.

### Consistency between spindle phase flows and spiral rotational dynamics

To demonstrate that the spatiotemporal dynamics of sleep spindles are largely organized by N2 spirals on a global scale, we introduce a consistency index that measures the similarity between spiral rotational dynamics and surrounding spindle phase flows. First, for each spiral center detected at each time step, we model an ideal phase velocity field that rotate around the spiral center, with phase velocity vectors always pointing at an angle perpendicular to the spiral center and consistent with its rotational direction. Next, we measure the absolute angle difference between the model and the phase velocity field observed in the hdEEG data. Finally, we select the phase velocity vectors with angle differences smaller than 45 degrees as consistent vectors and calculate the percentage of consistent vectors across the entire phase velocity field as the consistency index. We repeat this process across all detected N2 spirals and measure the mean consistency index for each subject. The same procedure is applied to the null model.

### Spiral wave angular speed

Following the detection of full spirals, we also study their rotational (angular) speed^27^. First, we select the center of a spiral as the anchor where we define a polar coordinate system. Next, we calculate the spiral’s instantaneous rotational angle as the angle between the polar axis (where the phase equal to *-π* or *π* in radians) and the horizontal direction (0 in radians). Finally, we calculate the instantaneous rotational (angular) speed of a spiral by comparing its instantaneous rotational angles between two subsequent time steps (2 ms per time step). This process can be described in Eq. (5):

5 $$\varOmega (t)=({\theta }_{t}-{\theta }_{t-\Delta t})/\Delta t$$

Where $\varOmega (t)$ denotes the angular speed of a spiral at time *t*, ${\theta }_{t}$ is the instantaneous rotational angle at time t, ${\theta }_{t-\Delta t}$ is the instantaneous rotational angle at the previous time step of *t*, $\Delta t$ is the time step size in millisecond.

### Linearly constrained minimum variance (LCMV) beamformer-based source reconstruction

To validate our observations of spiral waves at the sensor level, we employ a source reconstruction procedure using a LCMV beamformer, following the standard pipeline (ft_sourceanalysis) provided by FieldTrip (fieldtrip-20250318). Since we were unable to conduct MRI scans, which would allow the construction of precise head and source models for each subject, we adopt a generic source model (fieldtrip/template/anatomy/surface_pial_both.mat) and head model (fieldtrip/template/headmodel/ standard_bem.mat) included in the FieldTrip toolbox.

Considering the high resolution of the generic source model ( ~ 346k vertices), applying source reconstruction procedure across all ~346k vertices is very time consuming. To increase the efficiency of our procedure, we down-sample the 346k source model to 8k vertices via k-means clustering process (kmeans in Matlab) before source reconstruction and then interpolate the 8k source signals back to 346k to produce high resolution images. For precise interpolations over a convoluted cortical surface, it is desirable to perform linear interpolations across a flattened cortical map. Thus, we decide to swap the FieldTrip supplied generic source model with the generic source model (LR.pial.164k_fs_LR.surf.gii) from Human Connectome Project (HCP) database, which provides well-established, high-resolution flattened cortical surface maps. And to account for the difference in coordinate systems, we optimally align both source models via an iterative closest point (ICP) algorithm (pcregistericp in Matlab) followed by a Procrustes analysis (procrustes in Matlab).

Next, we construct the sensor model by optimally aligning the digitized sensor array to the generic head model via Affine transformation. We then compute the leadfield (ft_prepare_leadfield) based on the sensor model, source model and head model. Consistent with the standard procedure, we proceed to produce the covariance matrix (ft_timelockanalysis), followed by a spatial filter which is applied to the sensor level signals to generate source level signals across the cortical surface (ft_sourceanalysis).

It is worth noting that, although we conduct source reconstruction to validate our observation of spiral waves, most of our analysis are based on sensor level dynamics. To save processing time, the source-level projections of the spiral distribution in Figs. 2c, d and 3 are not directly measured from single-trial source level dynamics. Instead, we apply the population-averaged spatial filter to the sensor level spiral density map, which resembles a sensor-weighted source estimate of the spiral distribution. One exception is the exemplar snapshots shown in Fig. 2a, b, which represent the true single-trial output of the source reconstruction procedure.

### Spiral short-term and long-term consistency

We measure the short-term consistency of N2 spirals by calculating the similarity between the mean N2 spiral center distributions of each N2 epoch collected from the same overnight recording session. First, for each of the N2 epochs, we determine the mean spiral center distributions by counting how often they visit individual channel locations. This process thus produces a mean spiral center distribution map for each N2 epoch. We then calculate the cross-correlations between all possible pairs of these mean spiral center distribution maps across the overnight recording session. Finally, we calculate the mean correlation across all possible pairs and define it as spiral short-term consistency index. We repeat this process for each subject under either the baseline or treatment condition. This measurement thus quantifies the consistency of spiral center distributions within the same overnight recording session.

We measure the long-term consistency of N2 spirals in a similar manner. However, instead of calculating the similarity (correlation) between the mean spiral center distributions within the same recording session (either baseline or treatment), we compare the mean spiral center distributions between these two sessions (baseline Vs. treatment). After producing the mean spiral center distribution maps for each N2 epoch during either the baseline or treatment condition (using the same method as described above), we further average these maps to generate the mean spiral center distribution map for each subject under either the baseline or treatment condition. Finally, we calculate the cross-correlation between the subject-averaged spiral center distributions maps of baseline and treatment conditions and define it as spiral long-term consistency index. This process effectively measures how well the structure of spiral distributions are maintained across a three-month period separated by the CPAP therapy.

### Multiple linear regression

Multiple linear regression is conducted (‘regress’ function in MATLAB) with N2 epoch-based spiral short-term consistency, long-term consistency and their interaction terms (short-term consistency × long-term consistency) as independent variables and memory recall scores as the dependent variable.

### Null model

We generate the null model through a Fourier-based spatiotemporal phase shuffling procedure^27^. First, to avoid including NaN values outside the 2D cortical map, we select a maximal sized rectangular region within the *20×21* grid that contains no NaN values and apply a 3-dimensional (*x × y × t*) Fourier transform to the EEG dataset within the rectangular region using the ‘fftn’ function in MATLAB. This step computes the complex phasor at each point in the 3D dataset spanning space and time (x × y × t). Next, we multiply the complex phasor of each frequency component by a random phase factor, where the phase is uniformly sampled between −π and π. Finally, we apply an inverse Fourier transform to the shuffled data and reshape them back into a 3D surrogate data matrix of the original size. This process thus preserves both the spatial and temporal autocorrelations of the original signal while randomizing existing phase structures. We apply the same processing pipeline to the null model as the original hdEEG signals for spiral detection. Here, we adopt a conservative approach where only the spirals detected from the original signal with maximum radii larger than the 95^th^ percentile of the null distribution and a lifespan longer than three rotational cycles (228 ms on average) are retained for further analysis.

### Statistics and reproducibility

Details of the experiment and pre-processing procedures for study reproduction are available in the Methods section ‘Experimental design and data pre-processing’. We use both two-tailed paired-samples t-tests (using the ‘ttest’ function in MATLAB) and independent samples t-tests (using the ‘ttest2’ function in MATLAB) throughout our study. We adopt significance criteria of 0.05 followed by Benjamini-Hochberg FDR correction for multiple comparisons (via ‘bhfdr’ function in MATLAB).

### Reporting summary

Further information on research design is available in the Nature Portfolio Reporting Summary linked to this article.

## Supplementary information

Supplementary Information

Description of Additional Supplementary File

Supplementary Data

Supplementary Video 1

Supplementary Video 2

Supplementary Video 3

Supplementary Video 4

Reporting Summary

## Data Availability

The high-density EEG source data used in this study are not publicly available but can be accessed upon request. The source data underlying the figures can be found in Supplementary Data.

## Code availability

All MATLAB scripts used for conducting the analysis and generating example figures will be publicly available in a GitHub repository at the following URL: https://github.com/BrainDynamicsUSYD. These scripts can be used to reproduce the analysis and main figures presented in this study.
