# Supplementary material for: Spatiotemporal dynamics of sleep spindles form spiral waves that predict overnight memory consolidation and age-related memory decline
Source: Commun Biol. 2025 Jul 7;8:1014. doi: 10.1038/s42003-025-08447-4 (PMC12234817; doi:10.1038/s42003-025-08447-4)
Supplement: Supplementary file 1 — Supplementary Information [file 42003_2025_8447_MOESM1_ESM.pdf]

Supplementary Information for "Spatiotemporal dynamics of sleep spindles form spiral waves that predict overnight memory consolidation and age-related memory decline"

Yiben Xu[1], Alexander McInnes[1], Chien-Hui Kao[2], Angela D'Rozario[2,3], Jianfeng Feng[4], Pulin Gong[1*]

[1]School of Physics, University of Sydney, New South Wales, 2006, Australia

[2]CIRUS, Centre for Sleep and Chronobiology, Woolcock Institute of Medical Research, Macquarie University, New South Wales, Australia

[3]School of Psychological Sciences, Macquarie University, New South Wales, Australia

[4]Institute of Science and Technology for Brain-Inspired Intelligence, Fudan University, Shanghai, China

* pulin.gong@sydney.edu.au

Video 1. Single-trial example of simultaneously recorded phase field and phase velocity field of the original EEG signals and the corresponding null model. Left, sample single-trial instantaneous phase field overlayed with the phase velocity field. The colour map denotes instantons phase values (in radians). The black and white circles mark the detected center locations of the clockwise and anticlockwise spirals, respectively. The black solid lines denote the lower and upper boundaries of the maximal-sized rectangle used to generated the null model. Right, same as left, but the corresponding null model.

Video 2. Single-trial example of simultaneously recorded phase field, amplitude field, power field and center trajectory of a long-range travelling spiral. Left, exemplar single-trial instantaneous phase field. The colour map denotes phase values (in radians). The black dot and solid line mark the location and trajectory of a spiral center, respectively. Middle, Same as left, but of simultaneously recorded signal amplitude. The colour map denotes min-max normalized signal amplitude. Right, Same as left, but of simultaneously recorded sigma power. The colour map denotes min-max normalized analytic power.

Video 3. Single-trial example of simultaneously recorded phase field, amplitude field, power field and center trajectory of a local spiral. Left, exemplar single-trial instantaneous phase field. The colour map denotes phase values (in radians). The black dot and solid line mark the location and trajectory of a spiral center, respectively. Middle, Same as left, but of simultaneously recorded signal amplitude. The colour map denotes min-max normalized signal amplitude. Right, Same as left, but of simultaneously recorded sigma power. The colour map denotes min-max normalized analytic power.

Video 4. Exemplar side-by-side comparisons of spiral dynamics between the sensor level and cortical surface projections. Top left, exemplar instantaneous amplitude field projected onto the cortical surface (source level). The colour map denotes min-max normalized signal amplitude. Top right, exemplar simultaneously recorded instantaneous phase field projected onto the cortical surface

(source level). The colour map denotes phase values (in radians). Bottom left, Same as top left, but at the sensor level. Bottom right, Same as top right, but at the sensor level.

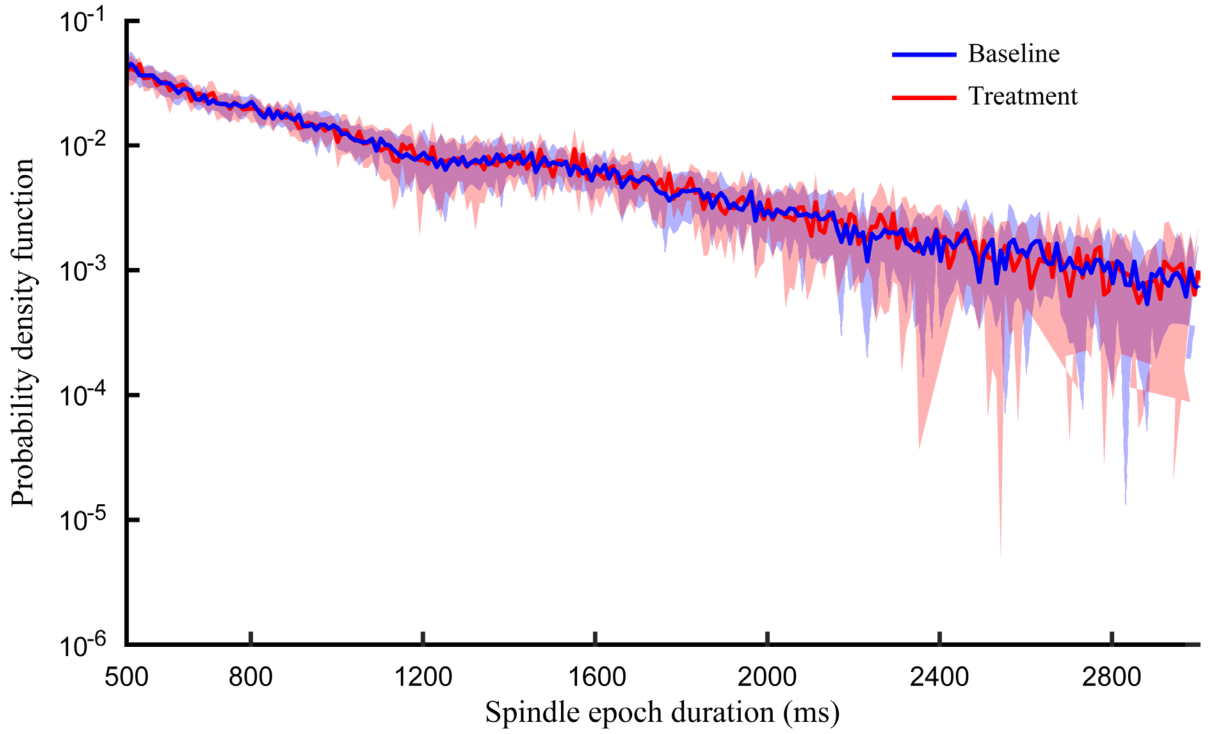

Fig. S1. Population-averaged probability density function of spindle epoch durations. Blue and red solid lines denote probability density function under baseline and treatment conditions, respectively. Shaded areas represent standard deviations from the mean.

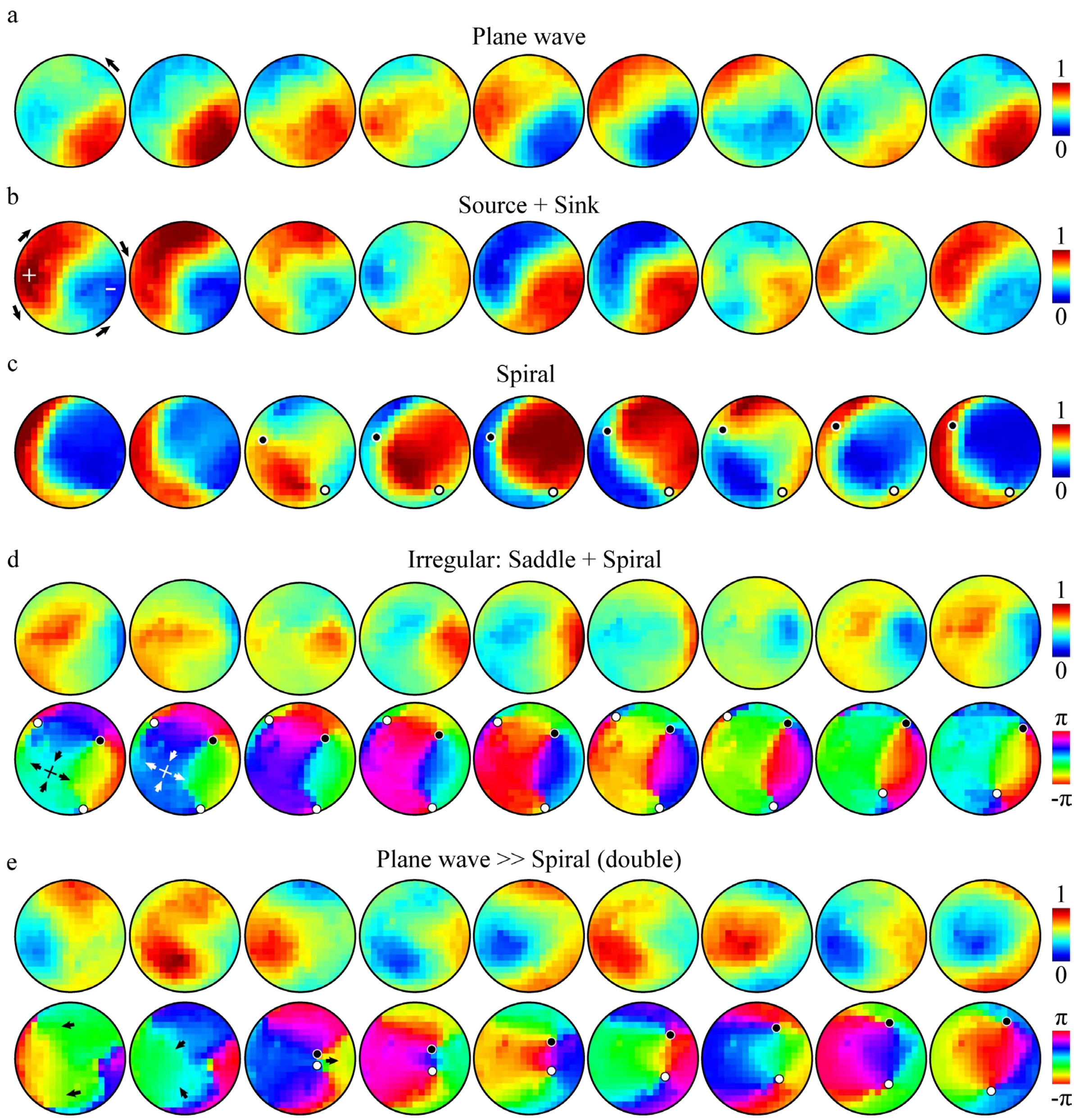

Fig. S2. Exemplar snapshots of different wave types and their interactions. a, Exemplar snapshots of a typical plane wave, each separated by 10ms in time. The colour scheme represents the min-max normalized signal amplitude, from 0 (trough, in blue) to 1 (peak, in red), respectively. **b**, Same as **a**, but for two source and sink patterns. The white '+' and '-' signs represent the source and sink points, respectively. The black arrows denote the propagation direction of the phase field. **c**, Same as **a**, but for a pair of spirals with opposite rotational directions. The black and white circles denote anticlockwise and clockwise rotating spirals, respectively. **d**, Exemplar snapshots of an irregular wave pattern consisted of three spirals and one saddle pattern, each separated by 20ms in time, in both the min-max normalized amplitude field (top) and the phase field (bottom). The colour schemes represent min-max normalized amplitude (top, 0: trough, 1: peak) and phase values (bottom, in radians), respectively. The black and white circles denote anticlockwise and clockwise rotating spirals, respectively. The black/white 'x' sign denote the saddle point. The four black/white arrows that are either pointing towards or away from the saddle point represent the forward and backward directions of the two eigenvectors at the saddle point (two arrows each for the stable and

unstable manifolds). **e**, Same as **d**, but illustrating the transition from a global plane wave to two counter-rotating spirals. The black arrows denote the propagation direction of the phase field.
