# Supplementary material for: Spatiotemporal dynamics of sleep spindles form spiral waves that predict overnight memory consolidation and age-related memory decline
Source: Commun Biol. 2025 Jul 7;8:1014. doi: 10.1038/s42003-025-08447-4 (PMC12234817; doi:10.1038/s42003-025-08447-4)
Supplement: Supplementary file 2 — Description of Additional Supplementary File [file 42003_2025_8447_MOESM2_ESM.pdf]

Description of additional supplementary file

File name: Supplementary Data
Description: Source data for figures

File name: Supplementary Video 1
Description: Single-trial example of simultaneously recorded phase field and phase velocity field of the original EEG signals and the corresponding null model. Left, sample single-trial instantaneous phase field overlayed with the phase velocity field. The colour map denotes instantons phase values (in radians). The black and white circles mark the detected center locations of the clockwise and anticlockwise spirals, respectively. The black solid lines denote the lower and upper boundaries of the maximal-sized rectangle used to generated the null model. Right, same as left, but the corresponding null model.

File name: Supplementary Video 2
Description: Single-trial example of simultaneously recorded phase field, amplitude field, power field and center trajectory of a long-range travelling spiral. Left, exemplar single-trial instantaneous phase field. The colour map denotes phase values (in radians). The black dot and solid line mark the location and trajectory of a spiral center, respectively. Middle, Same as left, but of simultaneously recorded signal amplitude. The colour map denotes min-max normalized signal amplitude. Right, Same as left, but of simultaneously recorded sigma power. The colour map denotes min-max normalized analytic power.

File name: Supplementary Video 3
Description: Single-trial example of simultaneously recorded phase field, amplitude field, power field and center trajectory of a local spiral. Left, exemplar single-trial instantaneous phase field. The colour map denotes phase values (in radians). The black dot and solid line mark the location and trajectory of a spiral center, respectively. Middle, Same as left, but of simultaneously recorded signal amplitude. The colour map denotes min-max normalized signal amplitude. Right, Same as left, but of simultaneously recorded sigma power. The colour map denotes min-max normalized analytic power.

File name: Supplementary Video 4
Description: Exemplar side-by-side comparisons of spiral dynamics between the sensor level and cortical surface projections. Top left, exemplar instantaneous amplitude field projected onto the cortical surface (source level). The colour map denotes min-max normalized signal amplitude. Top right, exemplar simultaneously recorded instantaneous phase field projected onto the cortical surface (source level). The colour map denotes phase values (in radians). Bottom left, Same as top left, but at the sensor level. Bottom right, Same as top right, but at the sensor level.
