# Supplementary material for: Spatiotemporal dynamics of sleep spindles form spiral waves that predict overnight memory consolidation and age-related memory decline
Source: Commun Biol. 2025 Jul 7;8:1014. doi: 10.1038/s42003-025-08447-4 (PMC12234817; doi:10.1038/s42003-025-08447-4)
Supplement: Supplementary file 8 — Reporting Summary [file 42003_2025_8447_MOESM8_ESM.pdf]

nature portfolio

Corresponding author(s): Pulin Gong

Last updated by author(s): 2025/06/15

Reporting Summary

Nature Portfolio wishes to improve the reproducibility of the work that we publish. This form provides structure for consistency and transparency in reporting. For further information on Nature Portfolio policies, see our Editorial Policies and the Editorial Policy Checklist.

Statistics

For all statistical analyses, confirm that the following items are present in the figure legend, table legend, main text, or Methods section.

n/a	Confirmed	
☐	☒	The exact sample size (*n*) for each experimental group/condition, given as a discrete number and unit of measurement
☐	☒	A statement on whether measurements were taken from distinct samples or whether the same sample was measured repeatedly
☐	☒	The statistical test(s) used AND whether they are one- or two-sided<br>*Only common tests should be described solely by name; describe more complex techniques in the Methods section.*
☐	☒	A description of all covariates tested
☐	☒	A description of any assumptions or corrections, such as tests of normality and adjustment for multiple comparisons
☐	☒	A full description of the statistical parameters including central tendency (e.g. means) or other basic estimates (e.g. regression coefficient) AND variation (e.g. standard deviation) or associated estimates of uncertainty (e.g. confidence intervals)
☐	☒	For null hypothesis testing, the test statistic (e.g. *F*, *t*, *r*) with confidence intervals, effect sizes, degrees of freedom and *P* value noted<br>*Give P values as exact values whenever suitable.*
☒	☐	For Bayesian analysis, information on the choice of priors and Markov chain Monte Carlo settings
☒	☐	For hierarchical and complex designs, identification of the appropriate level for tests and full reporting of outcomes
☐	☒	Estimates of effect sizes (e.g. Cohen's *d*, Pearson's *r*), indicating how they were calculated

Our web collection on statistics for biologists contains articles on many of the points above.

Software and code

Policy information about availability of computer code

Data collection	HydroCell Sensor Nets were used to collect high-density EEG data from a whole-head, 256-channel geodesic EEG system (Electrical Geodesics, Eugene, OR). EEG signals were amplified using NetAmps 300 amplifier and acquired/filtered with NetStation software (PhilipsNeuro EGI, Eugene, Oregon, USA).
Data analysis	EEGLAB and MATLAB 2020a; the code to reproduce the figures will be provided in the GitHub repository before publication: https://github.com/BrainDynamicsUSYD/.

For manuscripts utilizing custom algorithms or software that are central to the research but not yet described in published literature, software must be made available to editors and reviewers. We strongly encourage code deposition in a community repository (e.g. GitHub). See the Nature Portfolio guidelines for submitting code & software for further information.

Data

Policy information about availability of data

All manuscripts must include a data availability statement. This statement should provide the following information, where applicable:

- Accession codes, unique identifiers, or web links for publicly available datasets
- A description of any restrictions on data availability
- For clinical datasets or third party data, please ensure that the statement adheres to our policy

Data is available on request.

Research involving human participants, their data, or biological material

Policy information about studies with human participants or human data. See also policy information about sex, gender (identity/presentation), and sexual orientation and race, ethnicity and racism.

Reporting on sex and gender	All participants are males.
Reporting on race, ethnicity, or other socially relevant groupings	No information of race, ethnicity or other socially relevant groupings is collected.
Population characteristics	Nine male participants (age: 50.4 ± 6.5 years, apnea-hypopnea index/AHI: 51.7 ± 23.5/h, mean ± s.d., n = 9) with moderate-severe obstructive sleep apnea (OSA, AHI > 15).
Recruitment	Potential participants were identified by treating physicians at the Woolcock Institute of Medical Research sleep clinic, in Sydney, Australia, or from their research volunteer database of untreated OSA after having indicated their interest in being contacted for research projects.
Ethics oversight	The protocol was approved by the University of Sydney Human Research Ethics Committee (Project 2016/712) and the study was prospectively registered on the Australasian and New Zealand Clinical Trials Registry (ANZCTR) - https://www.anzctr.org.au ACTRN12617000336381. All participants provided written, informed consent prior to participation.

Note that full information on the approval of the study protocol must also be provided in the manuscript.

Field-specific reporting

Please select the one below that is the best fit for your research. If you are not sure, read the appropriate sections before making your selection.

☒ Life sciences ☐ Behavioural & social sciences ☐ Ecological, evolutionary & environmental sciences

For a reference copy of the document with all sections, see nature.com/documents/nr-reporting-summary-flat.pdf

Life sciences study design

All studies must disclose on these points even when the disclosure is negative.

Sample size	9 male participants with moderate-severe obstructive sleep apnea (OSA, AHI > 15).
Data exclusions	No data was excluded.
Replication	The main results were replicated on the same dataset by using different processing parameters/methods.
Randomization	No experimental conditions requiring randomization were applied in the study.
Blinding	Blinding is not relevant to this study because all participants are in a single group undergoing the same procedure.

Reporting for specific materials, systems and methods

We require information from authors about some types of materials, experimental systems and methods used in many studies. Here, indicate whether each material, system or method listed is relevant to your study. If you are not sure if a list item applies to your research, read the appropriate section before selecting a response.

Materials & experimental systems

n/a	Involved in the study	
☒	☐	Antibodies
☒	☐	Eukaryotic cell lines
☒	☐	Palaeontology and archaeology
☒	☐	Animals and other organisms
☐	☒	Clinical data
☒	☐	Dual use research of concern
☒	☐	Plants

Methods

n/a	Involved in the study	
☒	☐	ChIP-seq
☒	☐	Flow cytometry
☒	☐	MRI-based neuroimaging

Clinical data

Policy information about clinical studies
All manuscripts should comply with the ICMJE guidelines for publication of clinical research and a completed CONSORT checklist must be included with all submissions.

Clinical trial registration

The study was prospectively registered on the Australasian and New Zealand Clinical Trials Registry (ANZCTR) ACTRN12617000336381

Study protocol

Study protocol was approved by the University of Sydney Human Research Ethics Committee (Project 2016/712)

Data collection

The study was conducted at the Woolcock Institute of Medical Research, where participants visited a sleep laboratory equipped for high-density EEG studies. During the initial visit, they arrived at 5:00 pm, had dinner, and then filled out several questionnaires at 5:30 pm, including the Epworth Sleepiness Scale (ESS), Pittsburgh Sleep Quality Index, Depression Anxiety Stress Scale, and Insomnia Severity Index. At 6:00 pm, they completed a neurobehavioural performance test battery. Memory assessments were carried out both before and after an 8-hour sleep session, with lights turned off at 10:00 pm and turned on at 6:00 am. The learning phase of the memory tasks was conducted at 8:00 pm, approximately two hours before sleep, and the delayed recall phase took place at 7:00 am, about an hour after waking. These procedures were repeated three months later after participants underwent CPAP treatment. Throughout the study, participants maintained their usual sleep routines and avoided caffeine, alcohol, and daytime naps for 48 hours before and during data collection.

Outcomes

Primary outcome: Effectiveness of CPAP Treatment on Overnight Verbal Declarative Memory Consolidation.
This measure evaluates the impact of CPAP therapy on memory consolidation processes in patients with moderate to severe OSA. It is assessed by calculating the percent overnight recall, defined as the morning delayed recall score divided by the third trial evening recall score.
Secondary outcome: The Relationship Between the Spatiotemporal Dynamics of Sleep Spindles and Overnight Verbal Declarative Memory Consolidation.
This outcome examines how the characteristics of sleep spindles, particularly their spatiotemporal patterns, are associated with memory consolidation during sleep. It is assessed by calculating the correlation coefficient between feature indices of the spatiotemporal patterns of sleep spindles and the percent overnight recall, defined as the morning delayed recall score divided by the third trial evening recall score.

Plants

Seed stocks

Report on the source of all seed stocks or other plant material used. If applicable, state the seed stock centre and catalogue number. If plant specimens were collected from the field, describe the collection location, date and sampling procedures.

Novel plant genotypes

Describe the methods by which all novel plant genotypes were produced. This includes those generated by transgenic approaches, gene editing, chemical/radiation-based mutagenesis and hybridization. For transgenic lines, describe the transformation method, the number of independent lines analyzed and the generation upon which experiments were performed. For gene-edited lines, describe the editor used, the endogenous sequence targeted for editing, the targeting guide RNA sequence (if applicable) and how the editor was applied.

Authentication

Describe any authentication procedures for each seed stock used or novel genotype generated. Describe any experiments used to assess the effect of a mutation and, where applicable, how potential secondary effects (e.g. second site T-DNA insertions, mosiacism, off-target gene editing) were examined.
